# Phylogenetic spectrum and analysis of antibacterial activities of leaf extracts from plants of the genus *Rhododendron*

**DOI:** 10.1186/s12906-015-0596-5

**Published:** 2015-03-18

**Authors:** Ahmed Rezk, Jennifer Nolzen, Hartwig Schepker, Dirk C Albach, Klaudia Brix, Matthias S Ullrich

**Affiliations:** Molecular Life Science Research Center, Jacobs University Bremen, Campus Ring 1, Bremen, 28759 Germany; Institute for Biology and Environmental Sciences, Carl von Ossietzky University Oldenburg, Carl von Ossietzky Strasse 9-11, Oldenburg, 26111 Germany; Stiftung Bremer Rhododendronpark, Deliusweg 40, Bremen, 28359 Germany

**Keywords:** *Rhododendron*, Antimicrobial activity, Gram-positive bacteria, Multidrug efflux pump

## Abstract

**Background:**

Plants are traditionally used for medicinal treatment of numerous human disorders including infectious diseases caused by microorganisms. Due to the increasing resistance of many pathogens to commonly used antimicrobial agents, there is an urgent need for novel antimicrobial compounds. Plants of the genus *Rhododendron* belong to the woody representatives of the family *Ericaceae,* which are typically used in a range of ethno-medical applications. There are more than one thousand *Rhododendron* species worldwide. The Rhododendron-Park Bremen grows plants representing approximately 600 of the known *Rhododendron* species, and thus enables research involving almost two thirds of all known *Rhododendron* species.

**Methods:**

Twenty-six bacterial species representing different taxonomic clades have been used to study the antimicrobial potential of *Rhododendron* leaf extracts. Agar diffusion assay were conducted using 80% methanol crude extracts derived from 120 *Rhododendron* species. Data were analyzed using principal component analysis and the plant-borne antibacterial activities grouped according the first and second principal components.

**Results:**

The leaf extracts of 17 *Rhododendron* species exhibited significant growth-inhibiting activities against Gram-positive bacteria. In contrast, only very few of the leaf extracts affected the growth of Gram-negative bacteria. All leaf extracts with antimicrobial bioactivity were extracted from representatives of the subgenus *Rhododendron*, with 15 from the sub-section *Rhododendron* and two belonging to the section *Pogonanthum*. The use of bacterial multidrug efflux pump mutants revealed remarkable differences in the susceptibility towards *Rhododendron* leaf extract treatment.

**Conclusions:**

For the first time, our comprehensive study demonstrated that compounds with antimicrobial activities accumulate in the leaves of certain *Rhododendron* species, which mainly belong to a particular subgenus. The results suggested that common genetic traits are responsible for the production of bioactive secondary metabolite(s) which act primarily on Gram-positive organisms, and which may affect Gram-negative bacteria in dependence of the activity of multidrug efflux pumps in their cell envelope.

**Electronic supplementary material:**

The online version of this article (doi:10.1186/s12906-015-0596-5) contains supplementary material, which is available to authorized users.

## Background

For a long time, medicinal plants have been used as traditional treatments of a variety of human diseases. In many parts of the World, different kinds of plant material are still in use as a major source of traditional medicine formulations [1-3]. According to the World Health Organization, approximately 65% of the World’s populations integrate medicinal plants and products generated thereof into their primary health care strategies [4,5]. Importantly, in developing countries about 80% of the population is used to prepare traditional medicine formulation from plant sources [6].

Bacterial pathogens have developed different types of resistance to antimicrobial agents, thereby causing a significant increase in the costs of diagnostics and pharmaceutical treatments. Moreover, resistant microorganisms contribute to the currently observed dramatic increments in mortality and morbidity of patients affected by infectious diseases [7]. Since patients remain inflicted for a longer time period due to persisting microbial infection, the person-to-person transmission rates are prolonged and thus, enhanced. Increase in morbidity caused by antibiotics-resistant bacteria was recorded in several recent out-breaks such as pneumococcal infections, typhoid fever, and shigellosis in different regions world-wide [8]. The increasing resistance of human pathogens to commonly used antimicrobial agents motivated a renewed interest in the discovery of novel antimicrobial compounds. Several secondary metabolites of plants proved effective as biologically active agents against pathogens [9-11]. This is explained by the notion that plants acquired most of their secondary metabolite repertoire during evolution as metabolic byproducts, which then serve as defense compounds against predators such as insects and other herbivores, or against pathogens such as bacteria, fungi, or viruses [12,13].

*Rhododendron* L. (Ericaceae) is one of the largest genera of vascular plants and comprises eight subgenera with more than 1,000 species that populate habitats mostly in the Northern hemisphere [14]. Extracts of several species of *Rhododendron* are used in traditional medicine in the countries of their indigenous habitats. Among them are *R. ferrugineum* L.*, R. anthopogon* Don, or *R. tomentosum* (Stokes) Harmaja that are used for the treatment of inflammation, skin or gastrointestinal tract disorders, respectively [3,15-18]. These studies showed that the antibacterial activities of certain species of *Rhododendron* could be due to the presence of specific mono-, di-, or sesquiterpenoids, which had already been extracted from other plant families [19,20]. However, there is neither a comprehensive survey of the impressive number of chemical compounds in this genus nor have the later been tested systematically for their pharmacological potential [21]. The Bremen Rhododendron-Park possesses an unrivaled diversity of reliably identified *Rhododendron* species. Hence, the purpose of the current study was to comprehensively assess the spectrum of antibacterial activities of extracts prepared from 120 different *Rhododendron* species against a broad array of Gram-positive and Gram-negative bacteria. This study therefore aims to initiate the further identification and exploitation of novel plant-borne antibiotics derived from *Rhododendron* leaf extracts.

## Methods

### Plant material and extraction procedure

Fresh leaf material of 120 reliably identified *Rhododendron* species was collected from plants grown in the Rhododendron-Park Bremen (www.rhododendronparkbremen.de) from January 2012 to December 2013. Each plant species was sampled once without considering seasonal variations. The identities of all plant species have been verified according to the German Genebank Rhododendron Database provided by the Bundessortenamt (www.bundessortenamt.de/rhodo) (Additional file 1: Table S1). Material from all used plant species is publicly and freely available from the Rhododendron-Park Bremen upon request. The herein used *Rhododendron* species were chosen from five main subgenera: *Rhododendron*, *Hymenanthes*, *Tsutsusi*, *Pentanthera* and *Azaleastrum*, 10 sections, and 34 sub-sections in order to cover a broad phylogenetic spectrum of plants. Leaf material was immersed in liquid nitrogen and grinded to powder. Crude extracts were prepared by re-suspending 2 g (fresh weight) of leaf powder in 10 mL of the following solvents: 80% methanol (MeOH), ethyl acetate (EtOAc), or distilled water, respectively, each for 24 hours at 4°C. Non-dissolved leaf residues were removed by centrifugation (3,220 × g, 30 min, 4°C). The resulting supernatants were stored at-20°C, and the remaining, non-extracted powder was kept at-80°C for long-term storage to ensure reproducibilty of measurements from a standard reservoir of plant-derived material.

### Bacterial strains

**Figure 1:**
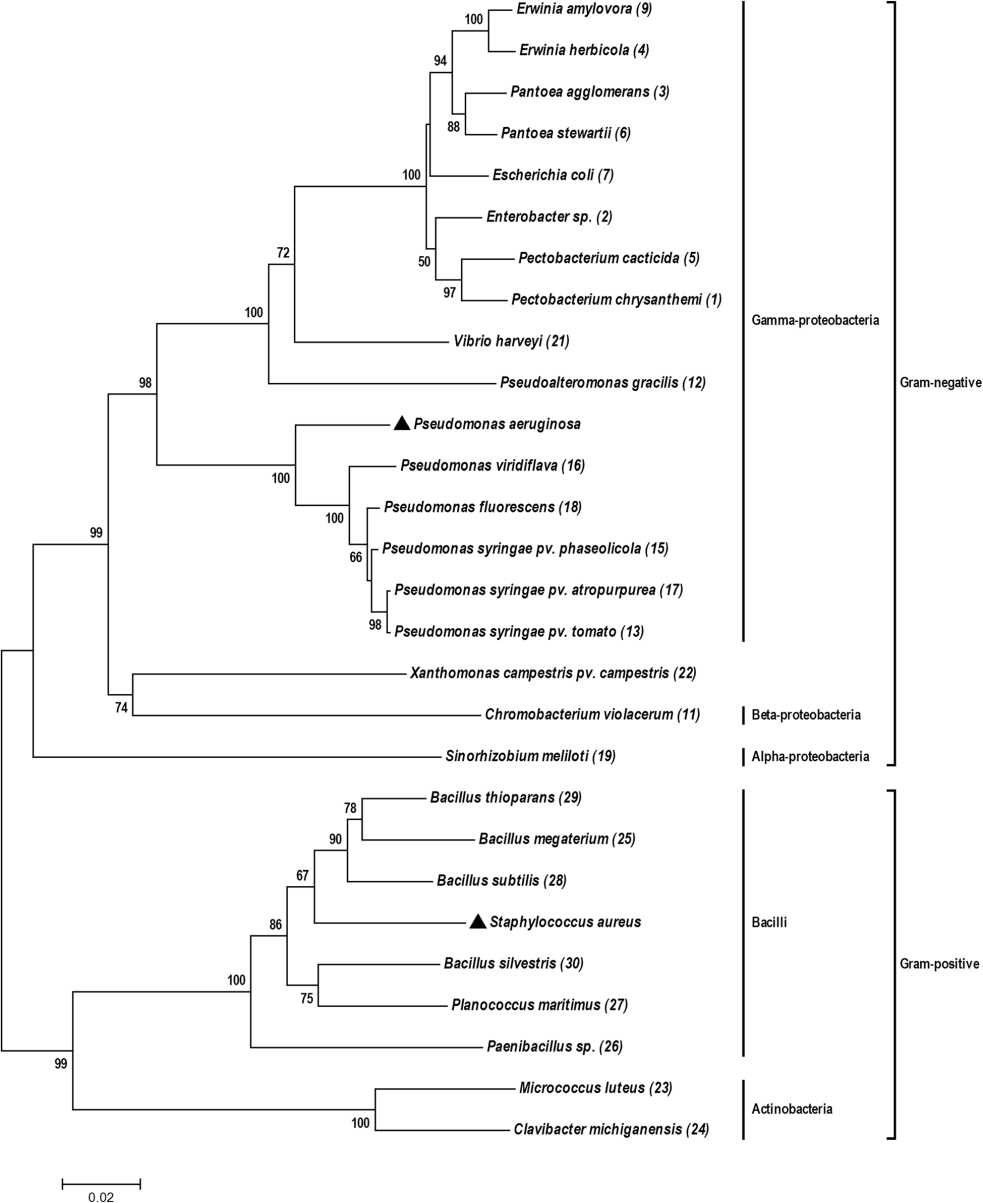
**Phylogenetic tree based on bacterial 16S rRNA gene sequences.** The phylogenetic tree was constructed using the neighbor-joining method showing the bacterial organisms (numbers in brackets) used in this study. Bootstrap values (1,000 replicates) lower than 50% are not shown. ▲ Filled triangles indicate the phylogenetic position of human pathogens which have not been used in this study. The scale bar 0.02 indicates 2% of nucleotide sequence substitution. The strain numbers are indicated in brackets.

Twenty-six bacterial species were randomly selected from different taxonomic clades to test for the susceptibility spectrum of crude extracts against a range of Gram-positive and Gram-negative bacteria (Figure 1). The phylogenetic tree was constructed by the Neighbor joining method in the Molecular Evolutionary Genetics Analysis (MEGA) software version 6 (Tamura, Stecher, Peterson, Filipski, and Kumar 2013). In order to investigate the role of multidrug efflux systems as bacterial defense mechanism, wild type and gene-specific mutants of the following bacterial species were analyzed: *Erwinia amylovora* 1189 (wild type), *Escherichia coli* TG1 (wild type) [22], *Pseudomonas syringae* DC3000 (wild type) [23] as well as the respective mutants with deletions in *acrAB, tolC*, or *mexAB* [24-28]. Additionally, knockout mutants of *E. amylovora* and *E. coli* with deletions in both, *acrAB* and *tolC* [25,27] were subjected to the antimicrobial analysis.

### Antimicrobial susceptibility test

Antimicrobial activity screening was conducted by the agar diffusion method [29]. Briefly, Lysogeny Broth (LB) agar plates were inoculated with 200 μL of the inoculum of the tester organism (1 × 10^7^ colony forming units per mL) by evenly spreading the cell suspensions over the agar surface. Holes with diameters of 5 mm were punched into the agar plates. Subsequently, 50 μL of the plant crude extracts were filled into each well. The plates were incubated overnight at 28°C or 37°C, according to the optimal growth temperature of each bacterial strain. Inhibition of microbial growth was determined by measuring the radius of the inhibition zone. For each bacterial strain, 80% methanol and 100% EtOAc solutions were used as negative solvent controls. All experiments were performed in triplicates and the results are presented as mean values.

### Principal component analysis and data analysis

Principal component analysis (PCA) as multivariate analysis of data was used to handle the multi-dimensionality of the data sets and to transform them into new, uncorrelated variables called principal components [30]. One hundred and twenty *Rhododendron* samples were tested against 26 bacterial strains (variables) using a random cross validation method. The matrix was designed by using the inhibition zone (mm). PCA was performed with The Unscrambler® 9.6 (CAMO AS, Oslo, Norway) software. All graphs were plotted using Origin (OriginLab, Northampton, MA).

## Results

### Bioactive metabolites and efficiency of solvent extraction

**Figure 2:**
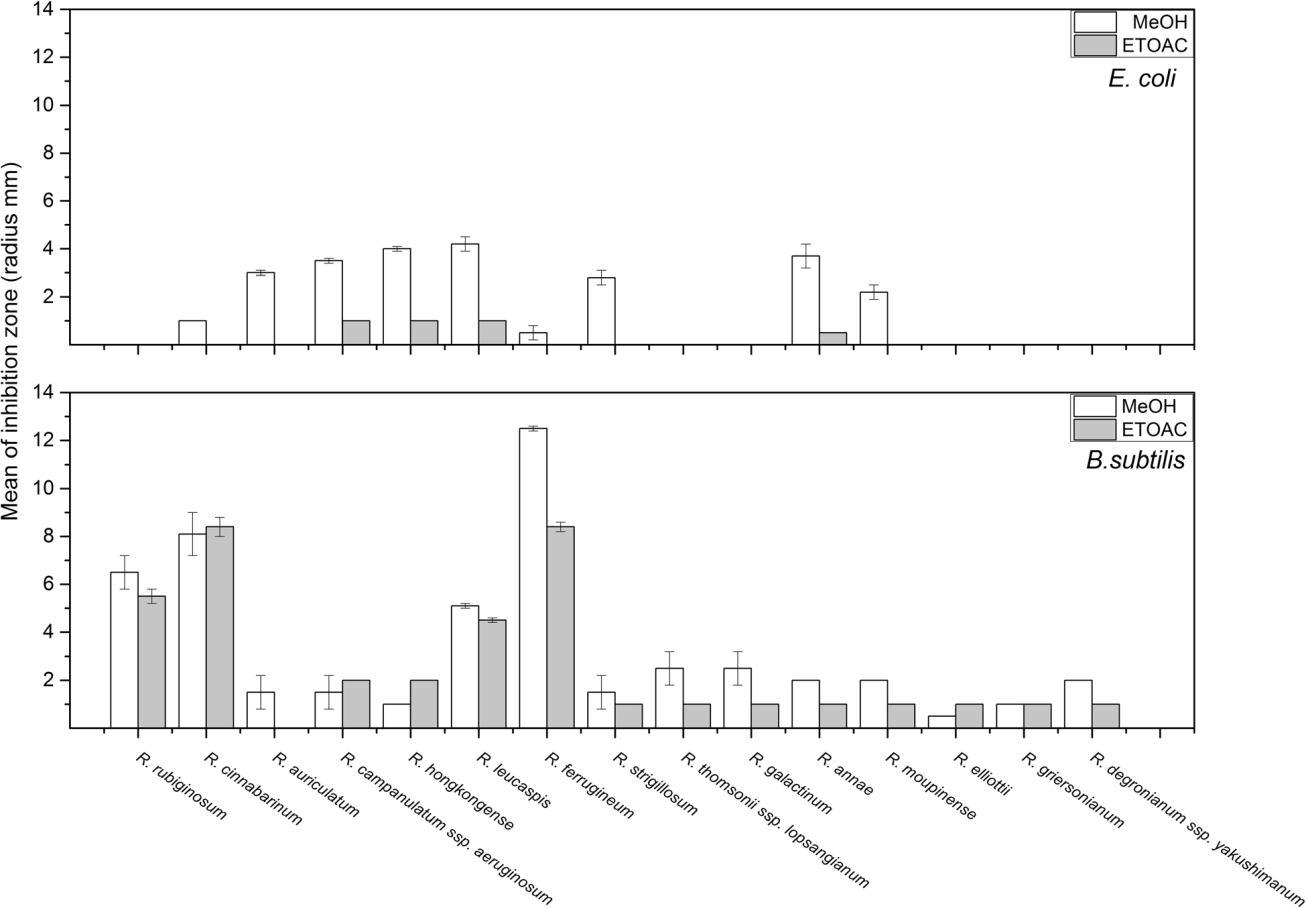
**Antimicrobial activities of methanol- and ethyl acetate-obtained crude leaf extracts of different** ***Rhododendron*** **species against** ***B. subtilis*** **and** ***E. coli.*** The radius of the inhibition zones was measured in triplicates and the values are given as means ± standard deviations. Treatment with solvents were used as negative controls and did not yield in inhibition zones (data not shown).

Initially, *Bacillus subtilis* 168 and *Escherichia coli* TG1 were used as model organisms for Gram-positive and Gram-negative bacteria, respectively. Crude leaf extracts from 120 *Rhododendron* species were obtained using different solvents in order to test for their efficacy to extract biologically active compounds from the plant material. The susceptibility of both bacterial model organisms was tested towards each of the crude leaf extracts. The antibacterial activity of *Rhododendron* leaf extracts obtained with MeOH or EtOAc as solvents was determined and categorized into three classes according to the radius of the inhibition zone: a) extracts of 13 *Rhododendron* species caused no inhibition, b) 71 extracts caused low inhibition (radius of less than 4 mm), and c) leaf extracts of 36 *Rhododendron* species caused inhibition (radius ranging from 4 to 12 mm) of growth of either *E. coli* or *B. subtilis* (Figure 2). None of the *Rhododendron* crude extracts obtained with distilled water showed any antibacterial activity (data not shown) indicating that bioactive compounds from *Rhododendron* are not readily water-soluble. The used solvent concentrations did not impact bacterial growth since treatment with MeOH and EtOAc alone did not induce inhibition zones (data not shown). In contrast, MeOH- and EtOAc-derived leaf extracts exhibited differential effects in that they were partially significantly active on both model organisms, suggesting that the contents or the concentration of potentially bioactive substances in the crude extracts might vary from one species of *Rhododendron* to the other. Interestingly, both tester organisms were generally more susceptible to treatment with the MeOH extracts while the EtOAc extracts showed less antimicrobial effects (Figure 2), indicating that methanol was more suitable to extracting bioactive compound(s) from powdered *Rhododendron* leaves. The extent by which the growth of the tester organisms was inhibited suggested a broad range of activities, which is possibly explained either by different compounds in various *Rhododendron* species or by diverse mechanisms of inhibition of bacterial growth mediated by the produced compounds.

### Spectrum of microbial susceptibility towards *Rhododendron* crude extracts

**Figure 3:**
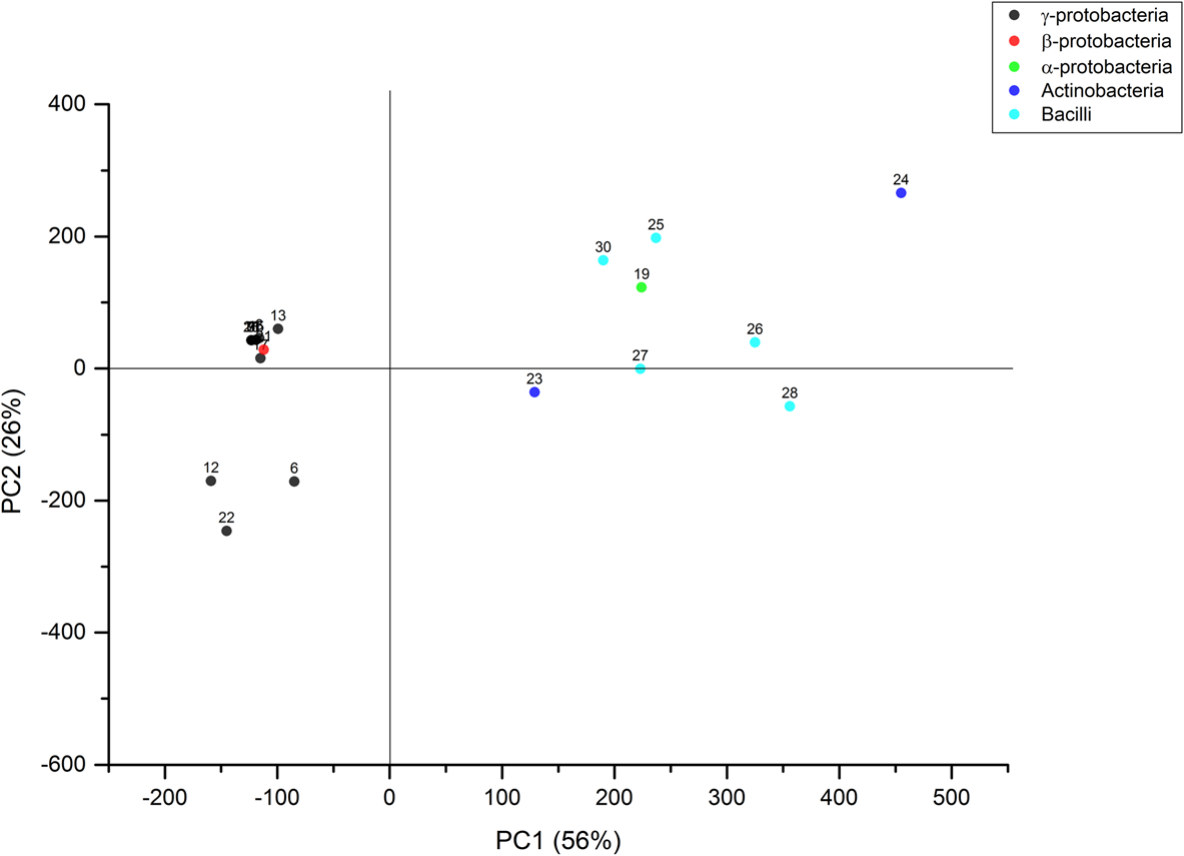
**PCA score plot.** Principal component analysis for score plot (random cross validation method) of the entire dataset (only *PC1* vs. *PC2* shown). Every dot represents one bacterial species and each color represents a particular group of bacterial organisms.

In order to test a wider spectrum of bacterial organisms, the antimicrobial activity test with all 120 *Rhododendron* crude extracts was extended to a set of 24 additional bacterial tester strains representing both, a broad phylogenetic spectrum as well as intra-genus diversity (Figure 1). As observed above, MeOH and EtOAc as solvents alone had no impact on growth of any of the tested bacterial organisms (data not shown). PCA was used to classify the spectrum of bioactivities of all *Rhododendron* extracts against all bacterial tester organisms according to their phylogenetic relatedness (Figure 3). The first and second principal components PC1 and PC2 explained 82% of the data indicating a high robustness of analysis. The data revealed that the microbial susceptibility towards *Rhododendron* extracts can be grouped into one group representing all Gram-positive tester organisms, while another group contained 17 out of 18 Gram-negative species (Figure 3) irrespective of the nature of the *Rhododendron* extract applied. The only Gram-negative species showing a similar susceptibility pattern as Gram-positive organisms was the alpha-protobacterium *Sinorhizobium meliloti*.

**Figure 4:**
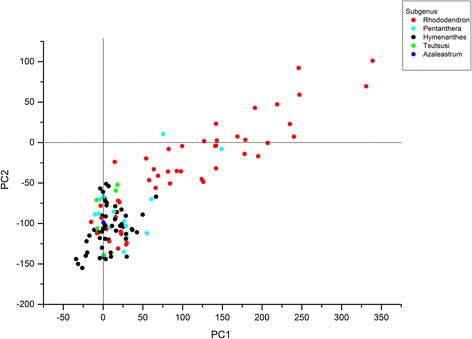
**PCA loading plot.** Principal component analysis for loading plot for 120 *Rhododendron* species (only *PC1* vs. *PC2* shown). Every dot represents one *Rhododendron* species and each color represents a subgenus of *Rhododendron*.

Five out of the 120 *Rhododendron* leaf extracts showed no growth inhibitory activity against any of the Gram-positive tester organisms: *R. elliottii* Watt ex Brandis, *R. hylaeum* Balfour & Farrer, *R. ponticum* L., *R. keiskei* Miquel, and *R. eriocarpum* (Hayata) Nakai (data not shown). These *Rhododendron* species belong to the subgenera *Hymenanthes* (three species), *Rhododendron*, and *Tsutsusi*, respectively. However, crude extracts obtained from 38 other *Rhododendron* species exhibited a moderate antimicrobial activity against 12 out of the 26 tested bacterial species. These plant species belonged to the subgenera *Azaleastrum*, *Hymenanthes*, *Rhododendron*, or *Tsutsusi*. The crude extracts derived from the remaining 77 *Rhododendron* species showed significant bioactivities against at least one of the 26 tester organisms. It is important to note that the spectrum of microbial susceptibilities towards *Rhododendron* extracts varied widely and showed dramatic differences, i.e. some of the tester organisms were susceptible towards leaf extracts from most of the *Rhododendron* species while other bacterial tester organisms were susceptible towards very few *Rhododendron* leaf extracts. *Bacillus thioparus* was the most sensitive tester species susceptible to all 77 potentially bioactive *Rhododendron* leaf extracts. The other tested Gram-positive bacteria showed similar susceptibility only towards leaf extracts derived from the 17 most bioactive *Rhododendron* species (Figure 4, Table 1).

**Table 1:** **List of** ***Rhododendron*** **species with the highest antimicrobial activities against Gram-positive bacteria**

Genebank-no.*	**Species name**	**Section**	**Sub-section**
100.345	*R. ferrugineum* L.	*Rhododendron*	*Rhododendron*
100.007	*R. ambiguum* Hemsley	*Rhododendron*	*Triflora*
2006/232	*R. anthopogon* Don ssp. *anthopogon* Betty Graham	*Pogonanthum*	*-*
NA	*R. hirsutum* L.	*Rhododendron*	*Rhododendron*
100.906	*R. anthopogon* ssp*. hypenanthum* Bale. F. & Cullen	*Pogonanthum*	*-*
100.326	*R. concinnum* Hemsley	*Rhododendron*	*Triflora*
100.881	*R. sichotense* Pojarkova	*Rhododendron*	*Rhodorastra*
100.322	*R. cinnabarinum* Hooker	*Rhododendron*	*Cinnabarina*
NA	*R. racemosum* Franchet	*Rhododendron*	*Scabrifolia*
100.882	*R. ledebourii* Pojarkova	*Rhododendron*	*Rhodorastra*
100.404	*R. rubiginosum* Franchet	*Rhododendron*	*Heliolepida*
100.474	*R. xanthostephanum* Merrill	*Rhododendron*	*Tephropepla*
101.048	*R. myrtifolium* Schott & Kotschy	*Rhododendron*	*Rhododendron*
100.370	*R. minus* Michaux	*Rhododendron*	*Caroliniana*
100.392	*R. polycladum* Franchet	*Rhododendron*	*Lapponica*
100.464	*R. spinuliferum* Franchet	*Rhododendron*	*Scabrifolia*
100.353	*R. hippophaeoides* var. *hippophaeoides* Hutchinson	*Rhododendron*	*Lapponica*

*Gene bank numbers used in the collection of the Rhododendron-Park Bremen.

NA: Not a plant of the German Genebank Rhododendron but a verified plant of the Rhododendron-Park Bremen.

In contrast, susceptibilities of the tested Gram-negative bacterial strains were classified into either low or moderate extent. Only one Gram-negative species, *Sinorhizobium meliloti*, belonging to the order of alpha-proteobacteria exhibited susceptibility to most of the bioactive *Rhododendron* extracts and was therefore similar in its susceptibility to the majority of Gram-positive bacteria (Figure 3, sample no. 19).

On one side, the PCA allowed classification of the bacterial tester organisms according to their degree of susceptibility after incubation with *Rhododendron* extracts. On the other hand, the analysis performed in this study allowed to successfully categorized the representatives of the genus *Rhododendron* into bioactive and non-bioactive species (Figure 4), where bioactivity is understood as antimicrobial activity as assayed by growth inhibition on LB-agar plates. The results illustrated that 41% of all analyzed species of the subgenus *Rhododendron* were highly bioactive against the majority of Gram-positive bacteria tested herein. In contrast, only two of the 14 species (14%) of the subgenus *Pentanthera* showed similarly high antimicrobial bioactivity. The majority of the remaining *Rhododendron* species representing other subgenera exhibited only moderate or low antimicrobial activities.

### Role of bacterial multidrug efflux pumps as potential resistance mechanisms

**Figure 5:**
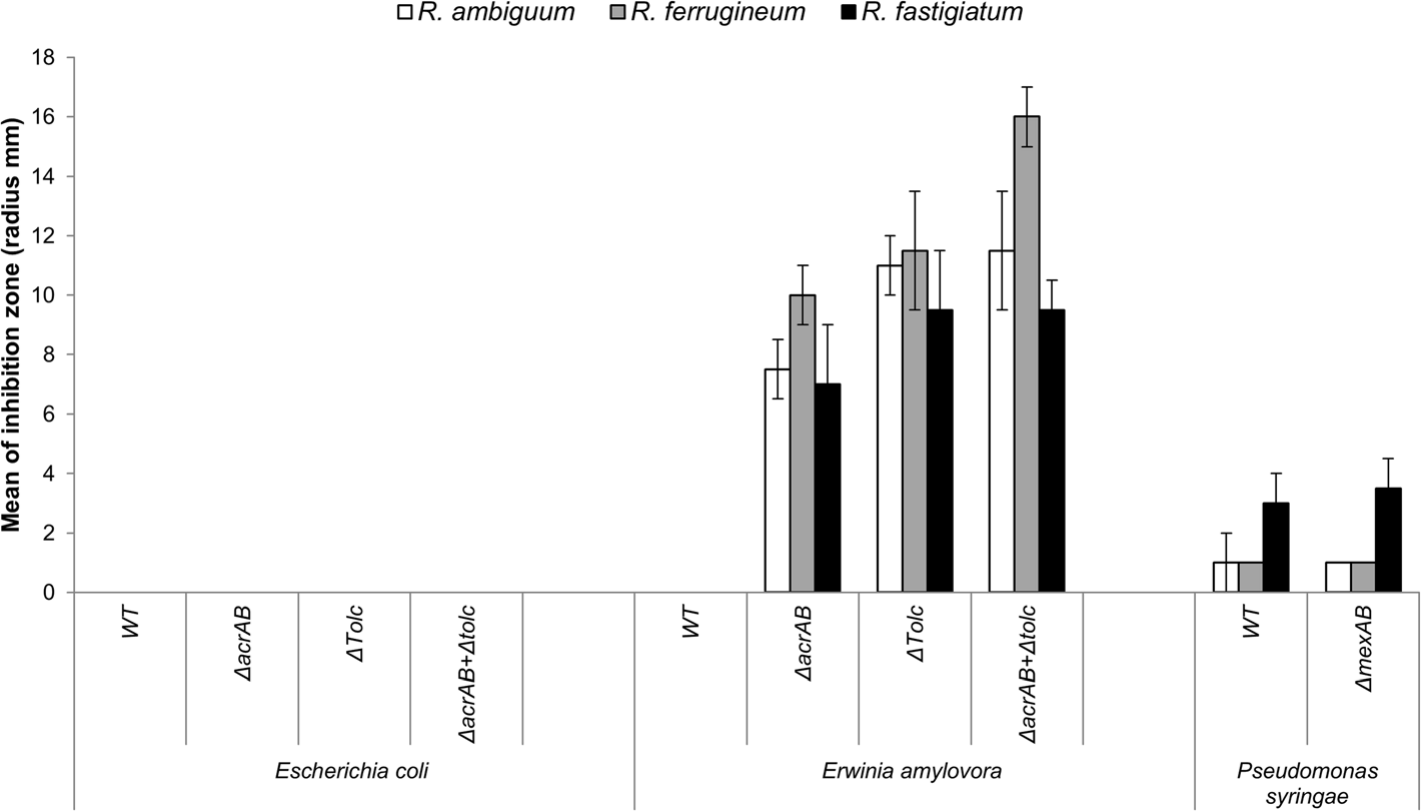
**Role of the multidrug efflux pump in Gram-negative bacteria.** Susceptibility tests for *E. coli*, *E. amylovora, P. syringae* and their respective RND-type multidrug efflux pump mutants to leaf extracts of three *Rhododendron* species effective against Gram-positive bacteria. WT, wild type. The radius of the inhibition zones was measured in triplicates and values are given as means ± standard deviations.

One important observation herein was the finding of higher susceptibility of Gram-positive over Gram-negative tester organisms towards *Rhododendron* leaf extracts, suggesting an easier passage of potential bioactive compounds into Gram-positive cells. In order to find out, whether one of the best studied antibiotics resistance mechanism, the RND-type multidrug efflux pump system of Gram-negative bacteria, is responsible for the lower susceptibility of the latter organisms, previously generated gene-specific knock-out mutants of *E. coli*, *Erwinia amylovora*, and *Pseudomonas syringae* with gene deletions in the multidrug efflux pump components *acr*AB, *tol*C, or *mex*AB were analyzed by treating them with crude extracts of *R. ambiguum* Hemsley, *R. ferrugineum* L., or *R. fastigiatum* Franchet (Figure 5). The *E. coli* wild type as well as all of its multidrug efflux pump mutants exhibited full resistance to all of the tested leaf extracts. However, data for the fire blight pathogen, *E. amylovora*, differed. While the wild type strain of this bacterium exhibited full resistance to each one of the tested *Rhododendron* extracts, single as well as double mutant strains with deletions of *tolC* and *acrAB* exhibited high sensitivity towards all three tested *Rhododendron* leaf extracts thereby rendering their phenotype to that of the Gram-positive bacteria (Figure 5). The *mexAB* multidrug efflux pump mutant of *P. syringae* did not show a significant reduction in growth when exposed to any of the *Rhododendron* leaf extracts.

In summary, these data suggested that some but not all of the multidrug efflux pump systems are likely to be directly involved in export of *Rhododendron*-derived bioactive antimicrobial compounds.

## Discussion

The purpose of this study was to conduct a comprehensive analysis of antibacterial activities of plants from the genus *Rhododendron*, which has the highest species richness among all woody plant genera [31]. Three different solvents were compared to optimize extraction efficiency for bioactive compounds from powdered *Rhododendron* leaf material. Our results demonstrated that methanol and ethyl acetate but not water extraction were suitable for this purpose. This finding is in agreement with those of previous studies, which reported that most of the bioactive secondary metabolites extracted from plant material are obtained by an aqueous methanol extraction [32].

The herein observed susceptibility of Gram-positive bacteria toward *Rhododendron* leaf extracts contrasted the results obtained with the majority of Gram-negative tester bacteria. This finding might be explained as follows: The cellular envelopes of either type of bacteria differ dramatically and the outer membrane of the Gram-negative cells with its lipopolysaccharide leaflet might pose an impermeable barrier for the methanol-extractable *Rhododendron*-derived bioactive compounds. Alternatively, various types of multidrug efflux pumps found in Gram-negative bacteria might extrude those bioactive compound(s). The latter hypothesis is strongly supported by findings of this study in that *E. amylovora* mutants with defects in the multidrug efflux pump system AcrAB/TolC were no longer resistant against treatment with *Rhododendron* leaf extracts. Similar conclusions have been drawn previously by our group with respect to treatment of *E. amylovora* with several chemical compounds originating from other plants [25,33,34]. Interestingly, multidrug efflux pump knock-out mutants of *E. amylovora* but not those of *E. coli* or *P. syringae* exhibted a significant growth inhibition when compared to the wild type strain upon exposure to *Rhododendron* extracts. These results indicated that presence of distinct multidrug efflux systems might explain the resistance towards some of the bioactive *Rhododendron* extracts. Since neither *E. coli* nor *P. syringae* mutants with the same defects exhibited growth inhibition upon exposure to these leaf extracts, additional factors must contribute to the resulting resistance in those organisms. This notion points to an interesting diversity of potential molecular mechanisms realized in different Gram-negative bacteria, thus providing some species with resistance towards bioactive compounds extracted from *Rhododendron* leaves while others will remain unaffected. In line with this conclusion, it has been described previously that the permeability of the outer membrane of *E. coli* differs from that of other Gram-negative microorganisms [25]. The diversity of potential resistance mechanisms among Gram-negative bacteria could also help explaining the unusual high susceptibility of *Sinorhizobium meliloti*. A future comparative multi-species genomic analysis is planned to shed light on the relationship of resistance and expression of individual genes among different Gram-negative bacterial species.

The herein studied bioactivities of *Rhododendron* leaf extracts showed a range of different effects towards the bacterial tester organisms possibly reflecting the phylogentic diversity of the analyzed *Rhododendron* species. The finding agrees with previously published results for *R. anthopogon* Don that exhibited antimicrobial activity against a group of Gram-positive bacteria (e.g. *Staphylococcus aureus*, *Enterococcus faecalis*, and *Bacillus subtilis*) [15]. We hypothesize that different *Rhododendron* genotypes may result in the formation of a broad range of compounds due to the diversity in structure and concentration of secondary metabolites resulting from different metabolic pathways. Previous authors had suggested that this might explain why certain plants become more or less susceptible to herbivores [35-37].

In contrast to the studies of others, which demonstrated that extracts from *R. setosum* Don, *R. ponticum*, *R. luteum* Sweet, *R. arboreum* Smith, *R. smirnowii* Trautvetter and *R. campanulatum* Don, inhibited the growth of *E. coli*, *B. subtilis* and *S. aureus* [18,38-41], corresponding extracts used in the current study did not show any antibacterial effects. This discrepancy might be attributed to a number of factors such as differences in compound extractability or in concentration of the secondary metabolites contained in the plants depending on the plants’ growing conditions with respect to variable ranges of biotic (e.g. herbivory, infection or allelopathy) or abiotic (e.g. nutrient, light, temperature and drought) stress factors. Some of the latter had been proven to trigger alterations of the secondary metabolite composition produced in one and the same plant species [42-46]. Taken together, we suggest that only a comprehensive and well-controlled analysis of the antimicrobial compound composition of plant-derived extracts will allow deciphering of the underpinning molecular and metabolic pathways, and only a delineation of genetically related phytomedical profiling will enable reproducible production of extracts which might then be useful as precursors for medicinal treatment options.

## Conclusions

The results obtained in this work revealed that 17 species of the genus *Rhododendron* exhibited antibacterial effects against Gram-positive bacteria. For the first time, a comprehensive study demonstrated that there is an accumulation of antimicrobial bioactivities among *Rhododendron* species of the subgenus *Rhododendron*. Consequently, a detailed phylogenetic assessment of the differences of various *Rhododendron* subgenera accompanied by in-depth phytochemical analysis of the extracts might shed light on the actual nature of the bioactive compound(s). Additionally, a further analysis of the bacterial susceptibility spectrum might indicate how the bioactive compound(s) act on certain microbes. Herein, potential resistance mechanisms of bacteria were shown to differ in a species-specific manner indicating the necessity to obtain an array of *Rhododendron*-derived bioactive compounds in order to inhibit a broad spectrum of potentially harmful bacteria.

## Additional file

Additional file 1: Table S1. List and origin of *Rhododendron* species tested in the study.

## Abbreviations

MeOH: Methanol
EtOAc: Ethyl acetate
PCA: Principal component analysis
