# Supplementary material for: Phylogenetic spectrum and analysis of antibacterial activities of leaf extracts from plants of the genus Rhododendron
Source: BMC Complement Altern Med. 2015 Mar 18;15:67. doi: 10.1186/s12906-015-0596-5 (PMC4367927; doi:10.1186/s12906-015-0596-5)
Supplement: Additional file 1: Table S1. — List and origin of Rhododendron species tested in the study. [file 12906_2015_596_MOESM1_ESM.docx]

**Additional file 1: Table S1**

List and origin of *Rhododendron* species tested in the study

| Genebank-No | Species Name | Subgenus | Section | Subsection |
| --- | --- | --- | --- | --- |
| 100.470 | *R.* *hongkongense* Hutchinson | *Azaleastrum* | *Azaleastrum* | *-* |
| 100.390 | *R.* *ovatum* (Lindley) Maximowicz | *Azaleastrum* | *Azaleastrum* |  |
| 100.886 | *R.* *moulmainense* Hooker | *Azaleastrum* | *Choniastrum* | *-* |
| 100.467 | *R.* *macabeanum* Watt Balfour | *Hymenanthes* | *Ponticum* | *Grandia* |
| 100.463 | *R.* *arboreum* ssp. *arboreum* Smith | *Hymenanthes* | *Ponticum* | *Arborea* |
| 100.799 | *R.* *wasonii* Hemsley & Wilson | *Hymenanthes* | *Ponticum* | *Taliensia* |
| 100.891 | *R.* *decorum* ssp. *diaprepes* (Balfour & Smith) Ming | *Hymenanthes* | *Ponticum* | *Fortunea* |
| 100.812 | *R.* *argyrophyllum* ssp. *nankingense* (Cowan) Chamberlain | *Hymenanthes* | *Ponticum* | *Argyrophylla* |
| 100.393 | *R.* *ponticum* L. | *Hymenanthes* | *Ponticum* | *Pontica* |
| 100.016 | *R.* *auriculatum* Hemsley | *Hymenanthes* | *Ponticum* | *Auriculata* |
| 100.784 | *R.* *campanulatum* ssp. *aeruginosum* (Hooker) Chamberlain | *Hymenanthes* | *Ponticum* | *Campanulata* |
| 100.420 | *R.* *strigillosum* Franchet | *Hymenanthes* | *Ponticum* | *Maculifera* |
| 100.491 | *R.* *thomsonii* ssp. *lopsangianum* Hooker | *Hymenanthes* | *Ponticum* | *Thomsonia* |
| 100.349 | *R.* *galactinum* Tagg | *Hymenanthes* | *Ponticum* | *Falconera* |
| 100.794 | *R.* *annae* Franchet | *Hymenanthes* | *Ponticum* | *Irrorata* |
| 100.496 | *R.* *elliottii* Watt ex Brandis | *Hymenanthes* | *Ponticum* | *Parishia* |
| 100.483 | *R.* *griersonianum* Balfour & Forrest | *Hymenanthes* | *Ponticum* | *Griersoniana* |
| 100.338 | *R.* *degronianum* ssp. *yakushimanum* (Nakai) Hara | *Hymenanthes* | *Ponticum* | *Pontica* |
| 100.417 | *R.* *smirnowii* Trautvetter | *Hymenanthes* | *Ponticum* | *Pontica* |
| 100.441 | *R.* *williamsianum* Rehder & Wilson | *Hymenanthes* | *Ponticum* | *Williamsiana* |
| 100.412 | *R.* *selense* ssp. *jucundum* (Balfour & Smith) Chamberlain | *Hymenanthes* | *Ponticum* | *Selensia* |
| 100.439 | *R.* *wardii* var. *puralbum* (Balfour & Smith) Chamberlain | *Hymenanthes* | *Ponticum* | *-* |
| 100.880 | *R.* *protistum* Balfour & Forrest | *Hymenanthes* | *Ponticum* | *Grandia* |
| 100.874 | *R.* *coriaceum* Franchet | *Hymenanthes* | *Ponticum* | *Falconera* |
| 100.003 | *R.* *adenogynum* Diels | *Hymenanthes* | *Ponticum* | *Taliensia* |
| 100.887 | *R.* *adenopodum* Franchet | *Hymenanthes* | *Ponticum* | *Argyrophylla* |
| 100.821 | *R.* *alutaceum* var. *iodes* (Balfour & Forrest) Chamberlain | *Hymenanthes* | *Ponticum* | *Taliensia* |
| 100.830 | *R.* *aganniphum* var. *flavorufum* ((Balfour & Forrest) Chamberlain | *Hymenanthes* | *Ponticum* | *Taliensia* |
| 100.824 | *R.* *beesianum* Diels | *Hymenanthes* | *Ponticum* | *Taliensia* |
| 100.791 | *R.* *bureavii* Franchet | *Hymenanthes* | *Ponticum* | *Taliensia* |
| 100.457 | *R.* *longesquamatum* Schneider | *Hymenanthes* | *Ponticum* | *Maculifera* |
| 100.015 | *R.* *aureum* Georgi | *Hymenanthes* | *Ponticum* | *Pontica* |
| 100.796 | *R.* *purdomii* Rehder & Wilson | *Hymenanthes* | *Ponticum* | *-* |
| 100.889 | *R.* *ririei* Hemsley & Wilson | *Hymenanthes* | *Ponticum* | *Argyrophylla* |
| 101.066 | *R.* *faberi* Hemsley | *Hymenanthes* | *Ponticum* | *Taliensia* |
| 100.018 | *R.* *balangense* Fang | *Hymenanthes* | *Ponticum* | *Grandia* |
| 100.466 | *R.* *arboreum* ssp. *delavayi* (Franchet) Chamberlain | *Hymenanthes* | *Ponticum* | *Arborea* |
| 100.856 | *R.* *thayerianum* Rehder & Wilson | *Hymenanthes* | *Ponticum* | *Argyrophylla* |
| 101.118 | *R.* *campanulatum* Don | *Hymenanthes* | *Ponticum* | *Campanulata* |
| 100.795 | *R.* *sherriffii* Cowan | *Hymenanthes* | *Ponticum* | *Thomsonia* |
| 1999/259 | *R.* *neriiflorum* Franchet | *Hymenanthes* | *Ponticum* | *Neriiflora* |
| 100.315 | *R.* *campylocarpum* ssp. *caloxanthum* (Balfour & Farrer) Chamberlain | *Hymenanthes* | *Ponticum* | *Campylocarpa* |
| 100.249 | *R.* *brachycarpum* Don ex Don ssp. *fauriei* (Franchet) Chamberlain | *Hymenanthes* | *Ponticum* | *Pontica* |
| 100.248 | *R.* *brachycarpum* ssp. *brachycarpum* Don ex Don | *Hymenanthes* | *Ponticum* | *Pontica* |
| 100.860 | *R.* *wasonii* Hemsley & Wilson | *Hymenanthes* | *Ponticum* | *Taliensia* |
| 100.473 | *R.* *falconeri* ssp. *eximium* (Nuttall) Chamberlain | *Hymenanthes* | *Ponticum* | *Falconera* |
| 100.394 | *R.* *praevernum* Hutchinson | *Hymenanthes* | *Ponticum* | *Fortunea* |
| 100.489 | *R.* *hylaeum* Balfour & Farrer | *Hymenanthes* | *Ponticum* | *Thomsonia* |
| 100.884 | *R.* *montroseanum* Davidian | *Hymenanthes* | *Ponticum* | *Grandia* |
| 100.837 | *R.* *macrophyllum* Don ex Don | *Hymenanthes* | *Ponticum* | *Pontica* |
| 100.355 | *R.* *insigne* Hemsley & Wilson | *Hymenanthes* | *Ponticum* | *Argyrophylla* |
| 100.456 | *R.* *austrinum* (Small) Rehder | *Pentanthera* | *Pentanthera* | *Pentanthera* |
| 100.363 | *R.* *luteum* Sweet | *Pentanthera* | *Pentanthera* | *Pentanthera* |
| 100.431 | *R.* *vaseyi* Gray | *Pentanthera* | *Pentanthera* | *-* |
| 100.408 | *R.* *schlippenbachii* Maximowicz | *Pentanthera* | *Pentanthera* | *-* |
| 100.005 | *R.* *albrechtii* Maximowicz | *Pentanthera* | *Pentanthera* | *Pentanthera* |
| 101.044 | *R.* *arborescens* (Pursh) Torrey | *Pentanthera* | *Pentanthera* | *Pentanthera* |
| 100.012 | *R.* *atlanticum* (Ashe) Rehder | *Pentanthera* | *Pentanthera* | *Pentanthera* |
| 101.046 | *R.* *prunifolium* (Small) Millais | *Pentanthera* | *Pentanthera* | *Pentanthera* |
| 100.391 | *R.* *periclymenoides* (Michaux) Shinners | *Pentanthera* | *Pentanthera* | *Pentanthera* |
| 101.045 | *R.* *cumberlandense* Braun | *Pentanthera* | *Pentanthera* | *Pentanthera* |
| 100.250 | *R.* *calendulaceum* (Michaux) Torrey | *Pentanthera* | *Pentanthera* | *Pentanthera* |
| 100.380 | *R.* *occidentale* (Torrey & Gray) Gray | *Pentanthera* | *Rhodora* | *Pentanthera* |
| X/1266 | *R.* *pilosum* (MICHX. ex LAM.) CRAVEN | *Pentanthera* | *Rhodora* | *-* |
| NA | *R.* *multiflorum* var. *purpureum* (MAKINO) CRAVEN | *Pentanthera* | *Sciadorhodion* | *-* |
| 100.397 | *R.* *prinophyllum* (Small) Millais | *Pentanthera* | *Sciadorhodion* | *Pentanthera* |
| 101.115 | *R.* *canadense* (Linneaus) Torrey | *Pentanthera* | *Sciadorhodion* |  |
| 100.007 | *R.* *ambiguum* Hemsley | *Rhododendron* | *Pogonanthum* | *Triflora* |
| 100.750 | *R.* *maddenii* ssp. *maddenii* Hooker | *Rhododendron* | *Pogonanthum* | *Maddenia* |
| 100.426 | *R.* *tomentosum* (Stokes) Harmaja | *Rhododendron* | *Rhododendron* | *Ledum* |
| 100.368 | *R.* *micranthum* Turczaninow | *Rhododendron* | *Rhododendron* | *Micrantha* |
| 100.353 | *R.* *hippophaeoides* var. *hippophaeoides* Hutchinson | *Rhododendron* | *Rhododendron* | *Lapponica* |
| 100.370 | *R.* *minus* Michaux | *Rhododendron* | *Rhododendron* | *Caroliniana* |
| 100.404 | *R.* *rubiginosum* Franchet | *Rhododendron* | *Rhododendron* | *Heliolepida* |
| 100.322 | *R.* *cinnabarinum* Hooker | *Rhododendron* | *Rhododendron* | *Cinnabarina* |
| 100.468 | *R.* *leucaspis* Tagg | *Rhododendron* | *Rhododendron* | *Boothia* |
| 100.345 | *R.* *ferrugineum* L. | *Rhododendron* | *Rhododendron* | *Rhododendron* |
| 100.374 | *R.* *moupinense* Franchet | *Rhododendron* | *Rhododendron* | *Moupinensia* |
| 100.329 | *R.* *davidsonianum* Rehder & Wilson | *Rhododendron* | *Rhododendron* | *Triflora* |
| 101.054 | *R.* *tapetiforme* Balfour & Kingdon-Ward | *Rhododendron* | *Rhododendron* | *Lapponica* |
| 100.384 | *R.* *russatum* Balfour & Forrest | *Rhododendron* | *Rhododendron* | *Lapponica* |
| 100.803 | *R.* *trichanthum* Rehder | *Rhododendron* | *Rhododendron* | *Triflora* |
| 100.882 | *R.* *ledebourii* Pojarkova | *Rhododendron* | *Rhododendron* | *Rhodorastra* |
| 100.377 | *R.* *nitidulum* var. *omeiense* Philipson & Philipson | *Rhododendron* | *Rhododendron* | *Lapponica* |
| 100.392 | *R.* *polycladum* Franchet | *Rhododendron* | *Rhododendron* | *Lapponica* |
| 100.449 | *R.* *yunnanense* Franchet | *Rhododendron* | *Rhododendron* | *Triflora* |
| 100.343 | *R.* *fastigiatum* Franchet | *Rhododendron* | *Rhododendron* | *Lapponica* |
| 100.362 | *R.* *lutescens* Franchet | *Rhododendron* | *Rhododendron* | *Triflora* |
| 100.464 | *R.* *spinuliferum* Franchet | *Rhododendron* | *Rhododendron* | *Scabrifolia* |
| 100.326 | *R.* *concinnum* Hemsley | *Rhododendron* | *Rhododendron* | *Triflora* |
| 100.484 | *R.* *genestierianum* Forrest | *Rhododendron* | *Rhododendron* | *Genestieriana* |
| 100.498 | *R.* *rigidum* Franchet | *Rhododendron* | *Rhododendron* | *Triflora* |
| 100.477 | *R.* *scabrifolium* var. *spiciferum* (Franchet) Cullen | *Rhododendron* | *Rhododendron* | *Scabrifolia* |
| 100.495 | *R.* *polylepis* Franchet | *Rhododendron* | *Rhododendron* | *Triflora* |
| 100.906 | *R.* *anthopogon* ssp. *hypenanthum* Bale. F. & Cullen | *Rhododendron* | *Rhododendron* | *-* |
| 100.474 | *R.* *xanthostephanum* Merrill | *Rhododendron* | *Rhododendron* | *Tephropepla* |
| 100.748 | *R.* *keysii* Nuttall | *Rhododendron* | *Rhododendron* | *Cinnabarina* |
| 100.848 | *R.* *bracteatum* Rehder & Wilson | *Rhododendron* | *Rhododendron* | *Heliolepida* |
| 2006/232 | *R.* *anthopogon* Don ssp. *anthopogon* Betty Graham | *Rhododendron* | *Rhododendron* | *-* |
| 100.009 | *R.* *amesiae* Rehder & Wilson | *Rhododendron* | *Rhododendron* | *Triflora* |
| 100.654 | *R.* *augustinii* ssp. *chasmanthum* Cullen | *Rhododendron* | *Rhododendron* | *Triflora* |
| NA | *R.* *hirsutum* L. | *Rhododendron* | *Rhododendron* | *Rhododendron* |
| 101.358 | *R.* *keiskei* Miquel | *Rhododendron* | *Rhododendron* | *Triflora* |
| 100.471 | *R.* *pleistanthum* Balfour ex Wilding | *Rhododendron* | *Rhododendron* | *Triflora* |
| 100.424 | *R.* *tatsienense* Franchet | *Rhododendron* | *Rhododendron* | *Triflora* |
| 1470.000 | *R.* *triflorum* Hooker | *Rhododendron* | *Rhododendron* | *Triflora* |
| 100.376 | *R.* *mucronulatum* Turczaninow | *Rhododendron* | *Rhododendron* | *Rhodorastra* |
| 2010/384 | *R.* *setosum* Don | *Rhododendron* | *Rhododendron* | *Lapponica* |
| 2002/1084 | *R.* *scopulorum* Hutchinson | *Rhododendron* | *Rhododendron* | *Maddenia* |
| 101.047 | *R.* *neoglandulosum* Harmaja | *Rhododendron* | *Rhododendron* | *Ledum* |
| 100.881 | *R.* *sichotense* Pojarkova | *Rhododendron* | *Rhododendron* | *Rhodorastra* |
| NA | *R.* *racemosum* Franchet | *Rhododendron* | *Rhododendron* | *Scabrifolia* |
| 101.052 | *R. auritum* Tagg | *Rhododendron* | *Rhododendron* | *Tephropepla* |
| 101.048 | *R.* *myrtifolium* Schott & Kotschy | *Rhododendron* | *Rhododendron* | *Rhododendron* |
| 100.403 | *R.* *mucronatum* (Blume) G. Don var. *ripense* (Makino) Wilson | *Tsutsusi* | *Brachycalyx* | *-* |
| 100.422 | *R.* *tashiroi* Maximowicz | *Tsutsusi* | *Tsutsusi* | *-* |
| 100.904 | *R.* *dilatatum* Miquel | *Tsutsusi* | *Tsutsusi* |  |
| 100.357 | *R.* *kaempferi* Planchon | *Tsutsusi* | *Tsutsusi* |  |
| 100.872 | *R.* *oldhamii* Maximowicz | *Tsutsusi* | *Tsutsusi* |  |
| 100.650 | *R.* *eriocarpum* (Hayata) Nakai | *Tsutsusi* | *Tsutsusi* |  |

NA : not a plant of the German Genebank *Rhododendron* but nevertheless a verified plant of the Rhododendron-Park Bremen
